# Recurrence of bacteremia and infective endocarditis according to bacterial species of index endocarditis episode

**DOI:** 10.1007/s15010-023-02068-x

**Published:** 2023-07-03

**Authors:** Lauge Østergaard, Marianne Voldstedlund, Niels Eske Bruun, Henning Bundgaard, Kasper Iversen, Mia Marie Pries-Heje, Katra Hadji-Turdeghal, Peter L. Graversen, Claus Moser, Christian Østergaard Andersen, Kirstine Kobberøe Søgaard, Lars Køber, Emil Loldrup Fosbøl

**Affiliations:** 1 The Heart Centre, Rigshospitalet, University of Copenhagen, Blegdamsvej 9, 2100 Copenhagen, Denmark; 2 Statens Serum Institut, Copenhagen, Denmark; 3 Department of Cardiology, Zealand University Hospital, Roskilde, Denmark; 4 Clinical Institutes, Copenhagen and Aalborg University, Aalborg, Denmark; 5 Department of Clinical Medicine, University of Copenhagen, Copenhagen, Denmark; 6 Department of Cardiology, Herlev‐Gentofte Hospital, University of Copenhagen, Copenhagen, Denmark; 7 Department of Clinical Microbiology, Rigshospitalet, University of Copenhagen, Copenhagen, Denmark; 8 Department of Immunology and Microbiology, University of Copenhagen, Copenhagen, Denmark; 9 Department of Clinical Microbiology, Amager-Hvidovre Hospital, Copenhagen, Denmark; 10 Department of Clinical Microbiology, Aalborg University Hospital, Aalborg, Denmark; 11 Department of Clinical Medicine, Aalborg University, Aalborg, Denmark; 12 Department of Clinical Epidemiology, Aarhus University Hospital, Aarhus, Denmark

**Keywords:** Infective endocarditis, Relapse, Recurrence, Reinfection, Bacteremia

## Abstract

**Purpose:**

In patients surviving infective endocarditis (IE) recurrence of bacteremia or IE is feared. However, knowledge is sparse on the incidence and risk factors for the recurrence of bacteremia or IE.

**Methods:**

Using Danish nationwide registries (2010–2020), we identified patients with first-time IE which were categorized by bacterial species (*Staphylococcus aureus, Enterococcus* spp., *Streptococcus* spp., coagulase-negative staphylococci [CoNS], ‘Other’ microbiological etiology). Recurrence of bacteremia (including IE episodes) or IE with the same bacterial species was estimated at 12 months and 5 years, considering death as a competing risk. Cox regression models were used to compute adjusted hazard ratios of the recurrence of bacteremia or IE.

**Results:**

We identified 4086 patients with IE; 1374 (33.6%) with *S. aureus*, 813 (19.9%) with *Enterococcus* spp., 1366 (33.4%) with *Streptococcus* spp., 284 (7.0%) with CoNS, and 249 (6.1%) with ‘Other’. The overall 12-month incidence of recurrent bacteremia with the same bacterial species was 4.8% and 2.6% with an accompanying IE diagnosis, while this was 7.7% and 4.0%, respectively, with 5 years of follow-up. *S. aureus*, *Enterococcus* spp., CoNS, chronic renal failure, and liver disease were associated with an increased rate of recurrent bacteremia or IE with the same bacterial species.

**Conclusion:**

Recurrent bacteremia with the same bacterial species within 12 months, occurred in almost 5% and 2.6% for recurrent IE*. S. aureus, Enterococcus* spp., and CoNS were associated with recurrent infections with the same bacterial species.

**Supplementary Information:**

The online version contains supplementary material available at 10.1007/s15010-023-02068-x.

## Introduction

Infective endocarditis (IE) is associated with high mortality and for survivors of IE, recurrence of infection is a feared complication [1, 2]. Eradication of the infection and identification of primary and secondary foci of the infection is the main priority in reducing the risk of a recurrent episode, however, eradication may be difficult as the bacteria may be coated within a fibrin-layered thrombus placed on valve tissue with low metabolic activity [3]. For these reasons, long-term antibiotics (2–6 weeks) are recommended and in some instances surgical intervention may be needed for complete eradication [4]. Despite this, studies suggest that 1–3% of patients, surviving the initial episode of IE, experience a recurrent episode within 6 months with the same bacterial species [5–8]. Traditionally, recurrent episodes of IE have been divided into relapse and reinfection [4]. An operational definition of relapse has been defined as a recurrent episode of IE with the same bacterial species as the index IE within 6 months while reinfection has been defined as a new IE episode with another bacterial species or the same bacterial species beyond 6 months from primary IE. However, some studies have shown that a period of 12 months could be used to identify relapses using molecular analysis [8, 9]. A recurrent episode of bacteremia with the same bacterial species as the primary IE episode may be seen as deficient eradication and this clinical measurement has been used in a randomized controlled trial for this assessment [10]. Knowledge of the microbiological etiology is an important parameter to guide treatment, follow-up and the identification of possible primary foci. The bacterial species may also assist expectations on prognosis. However, little data are present on how different bacterial species associate with the recurrence of bacteremia or IE. Prior studies examining risk factors for relapse have been limited by low numbers and low generalizability [7, 8, 11]. Therefore, we set forth to examine the associated rate of recurrence of bacteremia or IE with the same bacterial species within (i) 12 months, and (ii) 5 years from index IE. Further, we aimed to examine how bacterial species, as well as other patient factors, were associated with the recurrence of bacteremia or IE with the same bacterial species.

## Methods

### Data sources

Linkage of registries in Denmark is possible as every Danish citizen is provided with a unique identifier [12]. For the purpose of this study, we used the following registries: The Danish National Patient Registry, The Danish Cause of Death Registry, The Danish Population Registry, The Danish Prescription Registry, The Danish Microbiology Database. The Danish National Patient Registry provides information on every visit to the hospital (in-patient, out-patient, and emergency room visits). Diagnosis codes of the visit are provided according to the International Classification of Diseases (ICD) version 10. One single primary diagnosis code is mandatory, and several secondary diagnosis codes may be provided. The Danish Cause of Death Registry was used to identify the date of death. The Danish Population Registry provided information on the sex and date of birth. The Danish Prescription Registry holds information on every redeemed prescription from a Danish pharmacy. The Danish Microbiology Database holds information on every blood culture drawn and analyzed at one of the 11 Departments of Clinical Microbiology in Denmark since 2010 [13]. The registries are considered of high quality and have been described in detail previously [12, 14–16].

### Study population

The study population included all patients with first-time IE (ICD 10 codes: I33, I38, I398, Supplementary Table 1) hospitalized from 2010–2020 (ensuring a minimum of 1 year of follow-up for all included patients) from the Danish National Patient Registry with a minimum length of hospital stay of 14 days unless patients died within 14 days of hospitalization. The positive predictive value (PPV) of these ICD-codes has been estimated at 90% [17, 18]. Linkage of nationwide data of all blood cultures drawn in Denmark from 2010–2020 was possible using a unique personal identifier. The bacterial species of the IE episode was identified as a positive blood culture within 30 days of IE admission date and up until IE discharge. A hierarchy to detect the most likely bacterial species was made: (1) *Staphylococcus aureus*, *Enterococcus* spp., and *Streptococcus* spp. (Supplementary Table 2a), (2) coagulase-negative staphylococci (CoNS, Supplementary Table 2b), (3) other bacteria (‘Other’, Supplementary Table 2c). Patients with blood culture-negative IE, patients with positive blood cultures not identified at the species level, patients where blood cultures were not drawn, and patients not surviving index IE were excluded. In a sensitivity analysis, recurrence of CoNS bacteremia was only considered an endpoint in the case where at least two positive blood cultures with the same species were identified with 24 h apart within 7 days to reduce the risk of contamination.

### Outcome, follow-up, and covariates

The primary outcome of this study was recurrent bacteremia (including IE episodes) or IE with the same bacterial species within 12 months. Secondary outcomes were (i) recurrent episode of bacteremia and IE with the same bacterial species with up to 5 years of follow-up, (ii) to identify factors associated with a recurrent episode with the same bacterial species of (a) bacteremia with 12 months of follow-up, (b) bacteremia within 5 years, (c) IE with 12 months of follow-up, and (d) IE within 5 years (iii) to determine mortality rate with a maximum of (i) 12 months and (ii) 5 years. For the primary outcome, patients were followed from the date of discharge from the primary episode of IE and up until a recurrent episode of bacteremia (including IE) or IE with the same bacterial species, December 31 2021, or death, whichever came first. In a supplementary analysis, only recurrent bacteremia (without IE) was considered an endpoint and for this analysis patients with recurrent IE with the same bacteremia were censored at the date of IE admission.

For patients with a recurrent episode of bacteremia or IE with the same bacterial species as the primary IE episode, we examined the 1-year mortality rate.

Medical history was assessed by the Danish National Patient Registry using out-patient and inpatient discharge diagnoses prior to IE admission date, Supplementary Table 1. Diabetes mellitus was defined as either a diagnosis code given at a hospital or from a redeemed prescription of an antidiabetic drug, Supplementary Table 1.

### Statistics

Baseline characteristics are presented by groups of bacterial species of the primary episode of IE; categorical variables in counts and percentages and numerical variables with a median and 25 and 75 percentiles. Using the Aalen Johansen estimator, we calculated the cumulative incidence of a recurrent episode of bacteremia or IE with the same bacterial species as for the index IE episode considering death as a competing risk, at 12 months and 5 years of follow-up. We used cause-specific Cox regression models to compute adjusted hazard ratios (HR) including 95% confidence intervals (95% CI) comparing the rate of a recurrent episode of bacteremia or IE with the same bacterial species according to bacterial species of index IE. In the models investigating a recurrent episode of bacteremia or IE, we included: sex, age, calendar year, bacterial species of primary IE, prosthetic heart valve prior to index IE, cardiac implantable electronic device (CIED) prior to index IE, chronic renal failure, liver disease, diabetes mellitus, surgery during index IE. Covariates included in the models were selected a priori based on clinical knowledge. Mortality was depicted using 1-Kaplan Meier estimates for the index IE per bacterial species. The proportional hazard assumption was tested using Martingale’s residuals and log–log plots.

### Ethics

In Denmark, registry-based studies do not require approval from the Ethics Committee or informed consent by law [19]. The study is approved by the data responsible institute (Capital Region of Denmark—Approval number: P-2019-263) in accordance with the General Data Protection Regulation. During data management, all personal identifiers were anonymized and sub classifications with three or less patients were not reported to assure anonymization as by the rules of Statistics Denmark.

## Results

We identified 6238 patients with IE from 2010–2020 and excluded (i) 1,189 (19.1%) patients who died in-hospital, (ii) 32 patients in which blood cultures were not drawn (0.5%), (iii) 707 (11.3%) patients who had a blood culture-negative IE, (iv) 11 (0.2%) patients with candida IE, and (v) 213 (3.4%) where the bacteremia was not specified on species level. Thus, a total of 4086 patients with IE were included; 1374 with *S. aureus* (33.6%), 813 with *Enterococcus* spp. (19.9% of which 85.6% were *E. faecalis*), 1366 patients with *Streptococcus* spp. (33.4%), 284 patients with CoNS (7.0%), and 249 with ‘Other’ (6.1%), Supplementary Table 2a–c shows distribution on species-level. The median age, proportion of male patients, and proportion of patients with a prosthetic heart valve were highest among patients with IE caused by *Enterococcus* spp. (Table 1 showing baseline characteristics). The proportion of patients with chronic renal failure was more prevalent among patients with *S. aureus*-IE and CoNS-IE, Table 1. Liver disease was most prevalent among patients with *S. aureus*-IE, Table 1. Chronic obstructive pulmonary disorder, atrial fibrillation, and malignancy were more prevalent among patients with *Enterococcus *spp.-IE, Table 1. Surgery during index IE was highest among CoNS-IE (27.5%) while this was lowest among *Streptococcus *spp.-IE (15.9%), Table 1.

**Table 1:** Baseline characteristics

	Staphylococcus aureus, *N* = 1374		Enterococcus, *N* = 813		Streptococcus, *N* = 1366		CoNS, *N* = 284		Other, *N* = 249	
	*N*	(%)	*N*	(%)	*N*	(%)	*N*	(%)	*N*	(%)
Male	913	(66.4)	647	(79.6)	917	(67.1)	200	(70.4)	182	(73.1)
Age, median years (IQR)	69.1 (56.4–78.5)		76.3 (69.2–81.8)		72.4 (62.2–80.5)		70–8 (59.9–77.8)		69.8 (59.9–77.0)	
Calendar group										
2010–2012	284	(20.7)	161	(19.8)	274	(20.1)	66	(23.2)	58	(23.3)
2013–2015	378	(27.5)	243	(29.9)	346	(25.3)	74	(26.1)	62	(24.9)
2016–2018	410	(29.8)	253	(31.1)	458	(33.5)	89	(31.3)	70	(28.1)
2019–2020	302	(22.0)	156	(19.2)	288	(21.1)	55	(19.4)	59	(23.7)
IE admission length of stay, median days (IQR)	43 (31–52)		45 (38–53)		38 (30–47)		45 (32–53)		42 (30–49)	
Surgery during IE admission	218	(15.9)	164	(20.2)	363	(26.6)	78	(27.5)	58	(23.3)
Dialysis during IE admission	274	(19.9)	76	(9.3)	77	(5.6)	59	(20.8)	19	(7.6)
**Medical history**										
PCI	155	(11.3)	102	(12.5)	120	(8.8)	31	(10.9)	29	(11.6)
CABG	106	(7.7)	111	(13.7)	95	(7.0)	33	(11.6)	22	(8.8)
Chronic renal failure	265	(19.3)	91	(11.2)	95	(7.0)	47	(16.6)	20	(7.4)
Prosthetic heart valve	158	(11.5)	268	(33.0)	281	(20.6)	64	(22.5)	52	(20.9)
CIED	247	(18.0)	182	(22.4)	149	(10.9)	76	(26.8)	55	(22.1)
Diabetes mellitus	384	(27.9)	211	(26.0)	251	(18.4)	68	(23.9)	53	(21.3)
Heart failure	310	(22.6)	216	(26.6)	227	(16.6)	75	(26.4)	48	(19.3)
AMI	176	(12.8)	103	(12.7)	113	(8.3)	41	(14.4)	24	(9.6)
Afib	322	(23.4)	326	(40.1)	366	(26.8)	88	(31.0)	61	(24.5)
Stroke	175	(12.7)	136	(16.7)	130	(9.5)	38	(13.4)	29	(11.6)
Alcohol abuse	134	(9.8)	71	(8.7)	102	(7.5)	19	(6.7)	21	(8.4)
COPD	184	(13.4)	153	(18.8)	131	(9.6)	28	(9.9)	22	(8.8)
Malignancy	210	(15.3)	172	(21.2)	247	(18.1)	52	(18.3)	51	(20.5)
Liver disease	127	(9.2)	41	(5.0)	72	(5.3)	12	(4.2)	16	(6.4)
**Pharmacotherapy**										
Beta blockade	522	(38.0)	360	(44.3)	435	(31.8)	120	(42.3)	95	(38.2)
RASi	557	(40.5)	367	(45.1)	533	(39.0)	127	(44.7)	103	(41.4)
Aspirin	394	(28.7)	267	(32.8)	325	(23.8)	89	(31.3)	76	(30.5)
ADPi	152	(11.1)	108	(13.3)	129	(9.4)	36	(12.7)	11	(4.4)
OAC	314	(22.8)	336	(41.3)	403	(29.5)	99	(34.9)	89	(35.7)
Loop diuretics	452	(32.9)	323	(39.7)	371	(27.2)	89	(31.3)	60	(24.1)
Statin	517	(37.6)	390	(48.0)	503	(36.8)	116	(40.8)	83	(33.3)

*CoNS* coagulase negative staphylococci, *IQR* interquartile range, *IE* infective endocarditis, *PCI* percutaneous coronary intervention, *CABG* coronary artery bypass grafting, *CIED* cardiac implantable electronic device, *AMI* acute myocardial infarction, *Afib* atrial fibrillation, *COPD* chronic obstructive pulmonary disease, *RASi* renin angiotensin system inhibitor, *ADPi* adenosine di phosphate inhibitor, *OAC* oral anticoagulant

### Recurrent episode of bacteremia with the same bacterial species

**Fig. 1:**
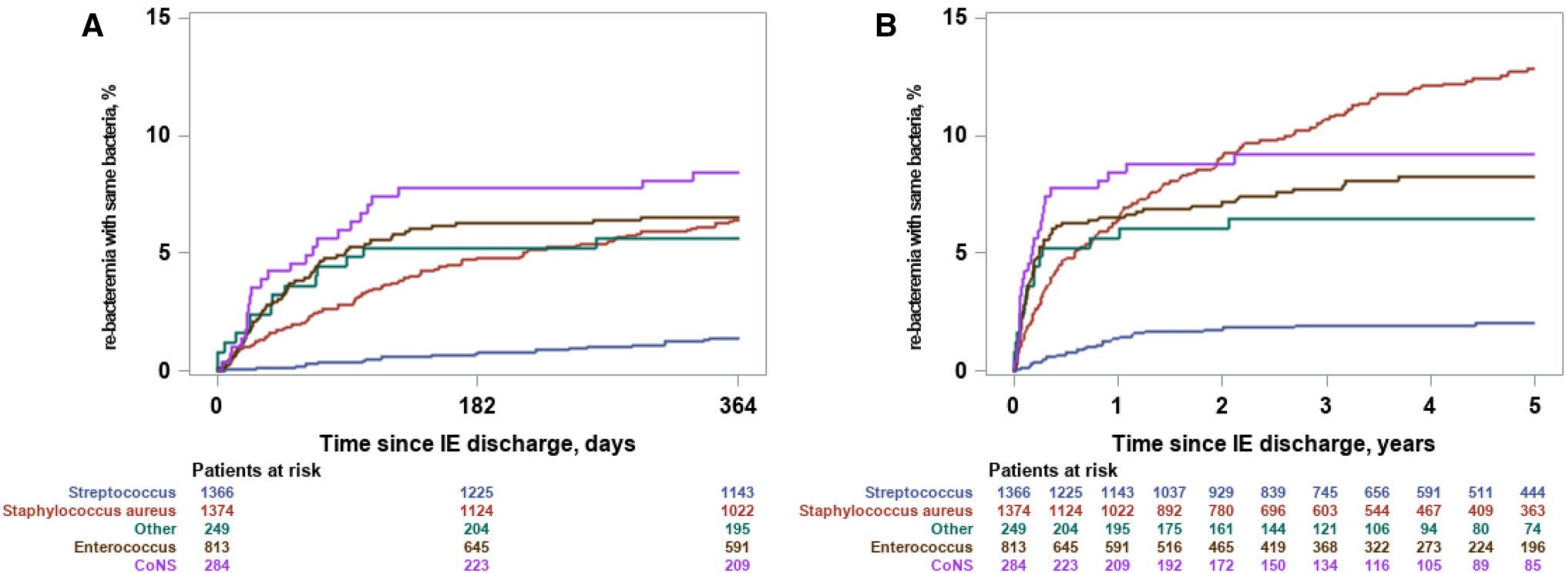
Cumulative incidence of recurrent bacteremia with the same bacterial species of the primary IE. The figure shows the cumulative incidence of a recurrent episode of bacteremia with the same bacterial species causing the primary episode of IE within 12 months of follow-up, panel **a** (left) and a maximum of 5 years of follow-up, panel **b** (right)

The overall cumulative incidence of recurrent bacteremia (including IE episodes) with the same bacterial species with a maximum of 12 months of follow-up was 4.8% (95% CI 4.2–5.5) (*n* = 198, Supplementary Table 3 shows distribution on species level). The cumulative incidence was 6.4% (95% CI 5.2–7.8%) for *S. aureus*, 6.5% (95% CI 5.0–8.4%) for *Enterococcus* spp., 1.4% (95% CI 0.9–2.1%) for *Streptococcus* spp., 8.5% (95% CI 5.6–12.1%) for CoNS, and 5.6% (95% CI 3.2–9.0%) for ‘Other’, Fig. 1a. With a maximum of 5 years of follow-up (median follow-up 2.9 years, 25 and 75 percentiles: 1.1–5.0), the overall cumulative incidence was 7.7% (95% CI 6.8–8.5) (*n* = 295, Supplementary Table 3 shows distribution on species level). The cumulative incidence was 12.8% (95% CI 11.0–14.8) for *S. aureus*, 8.2% (95% CI 6.4–10.3) for *Enterococcus* spp., 2.1% (95% CI 1.4–3.0) for *Streptococcus* spp., 9.2% (95% CI 6.2–12.9) for CoNS, and 6.5% (95% CI 3.9–10.0) for ‘Other’, Fig. 1b.

In a supplementary analysis of recurrence bacteremia without repeat IE, the same pattern was observed (Supplementary Fig. 1). Of note the majority of recurrent episodes of *Enterococcus* spp. bacteremia was accompanied by IE (Fig. 1a, b and Supplementary Fig. 1).

In a sensitivity analysis where recurrence of CoNS bacteremia was redefined as at least two positive blood cultures 24 h apart within 7 days, the incidence of recurrence CoNS bacteremia was 4.2% (95% CI 2.3–7.1) with a maximum of 12 months of follow-up and this was unchanged with a maximum of 5 years of follow-up (Supplementary Fig. 2).

### Recurrent episode of IE with same bacterial species

**Fig. 2:**
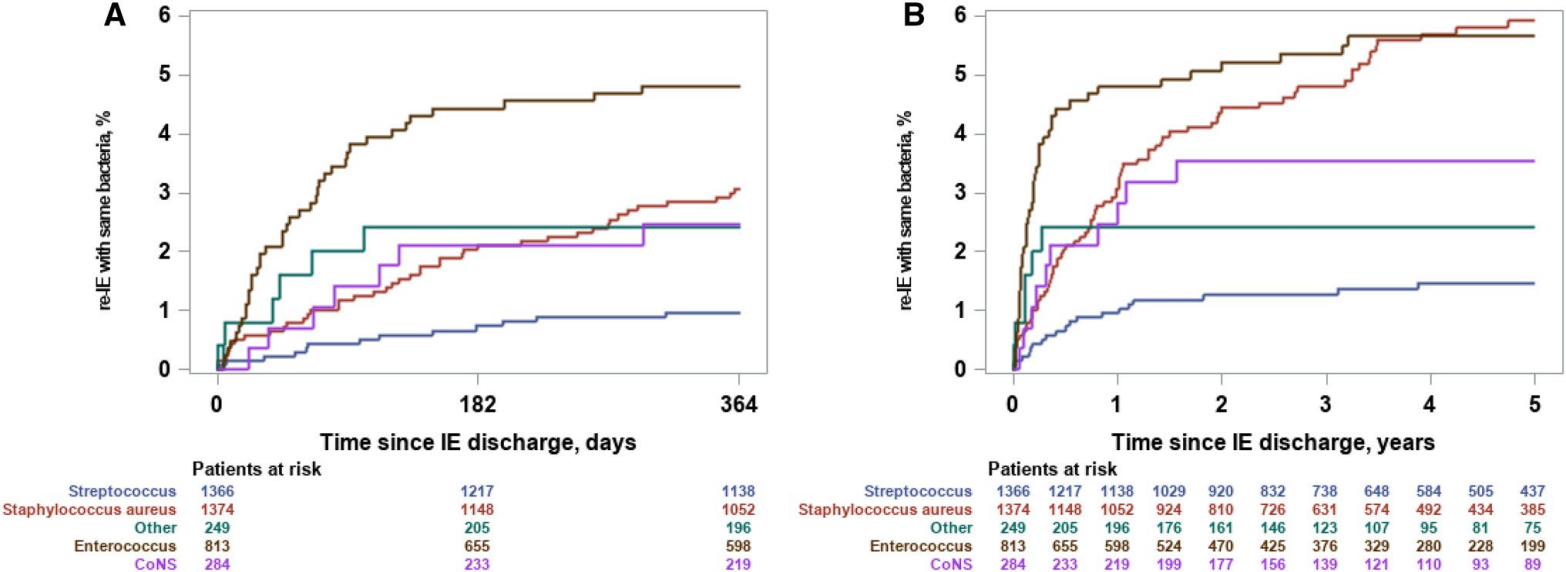
Cumulative incidence of recurrent IE with the same bacterial species. The figure shows the cumulative incidence of a recurrent episode of IE with the same bacterial species within 12 months of follow-up, panel **a** (left) and a maximum of 5 years of follow-up, panel **b** (right)

The overall cumulative incidence of a recurrent episode of IE with the same bacterial species within 12 months was 2.6% (95% CI 2.2–3.1) (*n* = 107, Supplementary Table 3 shows distribution on species level). The cumulative incidence was 3.1% (95% CI 2.2–4.1%) for *S. aureus*, 4.8% (95% CI 3.5–6.4%) for *Enterococcus* spp., 1.0% (95% CI 0.5–1.6%) for *Streptococcus* spp., 2.5% (95% CI 1.1–4.8%) for CoNS, and 2.4% (95% CI 1.0–4.9%) for ‘Other’, Fig. 2a. The overall cumulative incidence of a recurrent episode of IE within 5 years with the same bacterial species was 4.0% (95% CI 3.4–4.7) (*n* = 155, Supplementary Table 3 shows distribution on species level). The cumulative incidence was 5.9% (95% CI 4.7–7.4) for *S. aureus*, 5.7% (95% CI 4.2–7.4) for *Enterococcus* spp., 1.5% (95% CI 0.9–2.3) for *Streptococcus* spp., 3.6% (95% CI 1.8–6.2) for CoNS, 2.4% (95% CI 1.0–4.9) for ‘Other’, Fig. 2b.

### Factors associated with recurrent bacteremia and recurrent IE

**Fig. 3:**
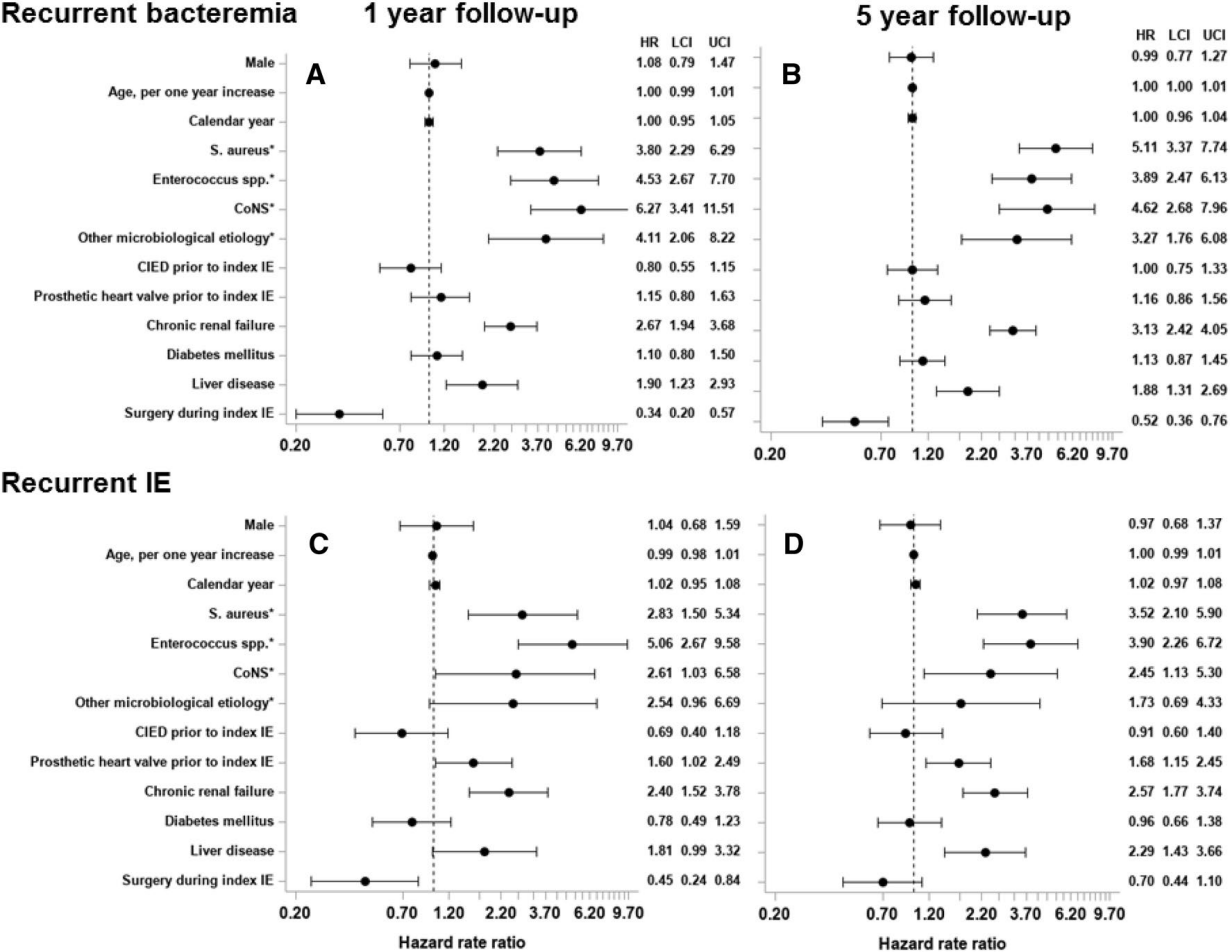
Factors associated recurrent bacteremia and IE. The figure shows the associated HR for recurrent bacteremia with the same bacterial species within 12 months (**a**) and 5 years of follow-up (**b**). The associated HR for recurrent IE with the same bacterial species with 12 months of follow-up (**c**) and 5 years of follow-up (**d**). *Streptococcus spp. as a reference group. *CoNS* coagulase-negative staphylococci, *CIED* cardiac implantable electronic device, *IE* infective endocarditis, *HR* hazard ratio, *LCI* lower confidence interval, *UCI* upper confidence interval

In the adjusted analysis, we found that patients with IE caused by *S. aureus, Enterococcus* spp., CoNS, and ‘Other’ were associated with a higher rate of a recurrent episode of bacteremia compared with *Streptococcus* spp. IE, Fig. 3a, b. *S. aureus*, *Enterococcus* spp., and CoNS were bacterial species associated with an increased rate of recurrent IE with the same bacterial species, across follow-up, as compared to patients with IE caused by *Streptococcus* spp., Fig. 3c, d. Chronic renal failure and liver disease were associated with an increased rate of both recurrent bacteremia and IE with the same bacterial species with a maximum of 1 and 5 years of follow-up, Fig. 3a, d. Surgery during index IE admission was associated with a decreased rate of recurrent bacteremia and IE with the same bacterial species at both 1 and 5 years of follow-up, Fig. 3a, d.

### Mortality

The 12-month mortality for the overall study population was 19.7% (*n* = 805); for *S. aureus* it was 21.7%, for *Enterococcus* spp. 23.1%, for *Streptococcus* spp. 15.4%, for CoNS 21.1%, and for ‘Other’ it was 19.3%. Within 5 years the overall mortality was 46.1% (*n* = 1668); for *S. aureus* it was 47.8%, for *Enterococcus* spp. 55.5%, for *Streptococcus* spp. 38.7%, for CoNS 46.9%, and for ‘Other’ it was 45.6%. For patients with recurrent bacteremia with the same bacterial species within 12 months of follow-up (*n* = 198), the 12-month mortality was 41.1%; for *S. aureus* it was 40.1%, for *Enterococcus* spp. 35.6%, for *Streptococcus* spp. 46.5%, for CoNS 39.8%, and for ‘Other’ it was 57.1%. For patients with recurrent IE with the same bacterial species within 12 months of follow-up (*n* = 107), the 12-month mortality was 38.1%; for *S. aureus* it was 42.8%, for *Enterococcus* spp. 29.4%, for *Streptococcus* spp. 35.9%, for CoNS 42.9%, and for ‘Other’ it was 50.0%.

## Discussion

Using nationwide data, we assessed the rate of recurrent bacteremia and IE with the same bacterial species in 4086 patients with IE. The study had three major findings. First, recurrent bacteremia with the same bacterial species was highest in patients with CoNS-IE with 12 months of follow-up, however within 5 years of follow-up the rate was highest among patients with *S. aureus*-IE. Second, for recurrent IE with the same bacterial species the rate was highest among patients with *Enterococcus* spp.-IE and *S. aureus*-IE. Third, chronic renal failure or liver disease was associated with an increased rate of recurrent bacteremia and IE with the same bacterial species within 12 months and 5 years of follow-up. Patients undergoing surgery for IE during index episode were associated with a lower rate of recurrent bacteremia and IE.

Few studies have investigated recurrence rate of bacteremia and IE following a primary episode of IE, however, some studies have examined the rate of recurrence for *S. aureus* bacteremia identifying rates of 5–17% within 90 days to 1 year [20–22]. Our estimates were lower, however, differences in the study population should be acknowledged. The rate of a recurrent IE with the same bacterial species has previously been examined, identifying a rate of a relapse (same bacterium within 6 months) between 0.9 and 2.2% with no detectable difference across bacterial species [4, 6, 7]. Our findings provide data underlining previous results with an overall recurrence rate of 2.6% with the same bacterial species within 12 months ranging from 1.0% in *Streptococcus* spp. to 4.8% in *Enterococcus* spp. For recurrence of bacteremia with the same bacterial species a shift in occurrence rate was seen across follow-up with CoNS having the highest rate of recurrence with 12 months of follow-up while this was *S. aureus*-IE with 5 years of follow-up. Recurrence of bacteremia seemed to reach a plateau for *E. faecalis*, CoNS and ‘Other’ within 6–12 months of follow-up while recurrence of *S. aureus* bacteremia continuously increased throughout the follow-up period. This may suggest that patients with *S. aureus*-IE have comorbidities or secondary foci that might increase the overall rate of recurrence. It must be noted that, especially for CoNS, there exist difficulties in differentiating infection from contamination, and in a sensitivity analysis where at least two positive blood cultures were needed, the incidence of recurrence of CoNS bacteremia was reduced from 7.4 to 4.2% with a maximum of 12 months of follow-up. These analyses provide estimates that indicate the range of recurrence of CoNS bacteremia that clinicians may integrate into patient information. Knowledge has previously been based upon data from multiple tertiary centers or single-center studies while our data brings knowledge from a generalizable population including data from nationwide registries [5, 6, 9]. These are important data to provide patients and clinicians with valid absolute estimates to understand the magnitude of the problem. Moreover, this may help future studies in the computation of valid event rates.

We identified that chronic renal failure or liver disease was associated with an increased rate of recurrence of bacteremia and IE. It was established decades ago that chronic renal failure is associated with an increased rate of IE [23] and some studies have also identified that this condition is associated with an increased rate of relapse [6] while other studies have not been able to detect this association [7]. This finding suggests the need for careful prophylactic measures in this subgroup of patients at high risk of a recurrent episode of bacteremia and IE—a complication with mortality within 12 months around 40%. Our data provide evidence that patients with *Enterococcus* spp.-IE are associated with the highest rate of a recurrent episode of IE which may help increase clinical awareness in optimal eradication of the infection among this subgroup of patients with IE. Further, a recurrent episode of bacteremia with the same bacterial species may be related to deficient eradication of primary and secondary foci (e.g. malignancy) and an extensive diagnostic work-up in this patient group may be important to help guide optimal treatment strategy for this patient group. Future studies could address this issue by different approaches, e.g. by assessment of the changes in the relapse rates by systematic uroscopy or colonoscopy in patients with *Enterococcus faecalis*-IE [4]. Further, we found that patients who underwent surgery during index IE admission had an up to 50% reduced rate of recurrent episode of IE, however, it must be noted that patients with prosthetic material have been associated with an increased rate of IE as compared with a matched background population. It must be acknowledged that the observational nature of this study may confound the lower associated rate of recurrence in patients undergoing surgery since this is a highly selected patient population.

### Limitations

Our study had some limitations. First, the identification of the study population is derived from hospital codes, which rely on the validity of the diagnosis codes. IE diagnosis codes from the Danish National Patient Registry were previously validated and found to have a positive predictive value of 90%, however, data on microbiological etiology were not included in the validation [17]. In this study, we were able to crosslink administrative, hospital registries with laboratory data on all blood cultures in Denmark from 2010–2021, excluding patients without blood cultures which likely further increased the PPV of the codes. From the assessment of IE from ICD-10 codes it was possible to differentiate between left- or right-sided IE. Further, we could not differentiate native valve endocarditis from prosthetic valve endocarditis or CIED-endocarditis. Second, we were not able to include patients from before 2010 as data from the departments of clinical microbiology was not available in a national database. Third, from the registries used data on echocardiography, detailed surgical data, laboratory values, valve cultures (for patients undergoing surgery), and antibiotic treatment were not available. Further, data on the cause of death was not available throughout the entire study period, hence the cause of death could not be assessed in detail. With this limitation in mind, our estimates (differences between microbiological etiologies in index IE) must be seen as conservative estimates. Fourth, the observational nature of the study precludes that no causal relations can be made, only associations. Fifth, data from the Danish Microbiology Database do not provide the opportunity to link blood cultures on a molecular level and differentiating relapse from recurrence of the same strain type from molecular standards was not available.

In conclusion, in patients with IE, recurrent bacteremia with the same bacterial species within 12 months occurred in almost 5% and recurrent IE with the same bacterial species occurred in 2.6%. Within 5 years of follow-up, these estimates were 7.7% for recurrent bacteremia and almost 5% for recurrent IE. *S. aureus, Enterococcus* spp., and CoNS were associated with the highest rate of recurrent bacteremia and IE. Chronic renal failure and liver disease were factors associated with an increased rate of recurrent bacteremia and IE across follow-up. Surgery during index IE was associated with a lower rate of recurrent bacteremia and IE. Our data underline the need for optimal eradication of infection foci and prophylactic measures in patients with IE—especially for patients with *Enterococcus* spp.-IE, *S. aureus*-IE and in patients with chronic renal failure or liver disease.

### Supplementary Information

Below is the link to the electronic supplementary material.

Supplementary file1 (PDF 127 KB)

Supplementary file2 (PDF 133 KB)

Supplementary file3 (DOCX 30 KB)

## Data Availability

Data is owned by a third party and the authors’ do not have the right to share data, however, the authors are willing to help acquire the necessary approvals for data sharing.
