# Supplementary material for: Recurrence of bacteremia and infective endocarditis according to bacterial species of index endocarditis episode
Source: Infection. 2023 Jul 3;51(6):1739–47. doi: 10.1007/s15010-023-02068-x (PMC10665237; doi:10.1007/s15010-023-02068-x)
Supplement: Supplementary file 1 — Supplementary file1 (PDF 127 KB) [file 15010_2023_2068_MOESM1_ESM.pdf]

Supplementary Figure 1. Cumulative incidence of recurrent bacteremia (without IE) with the same bacterial species as the primary IE

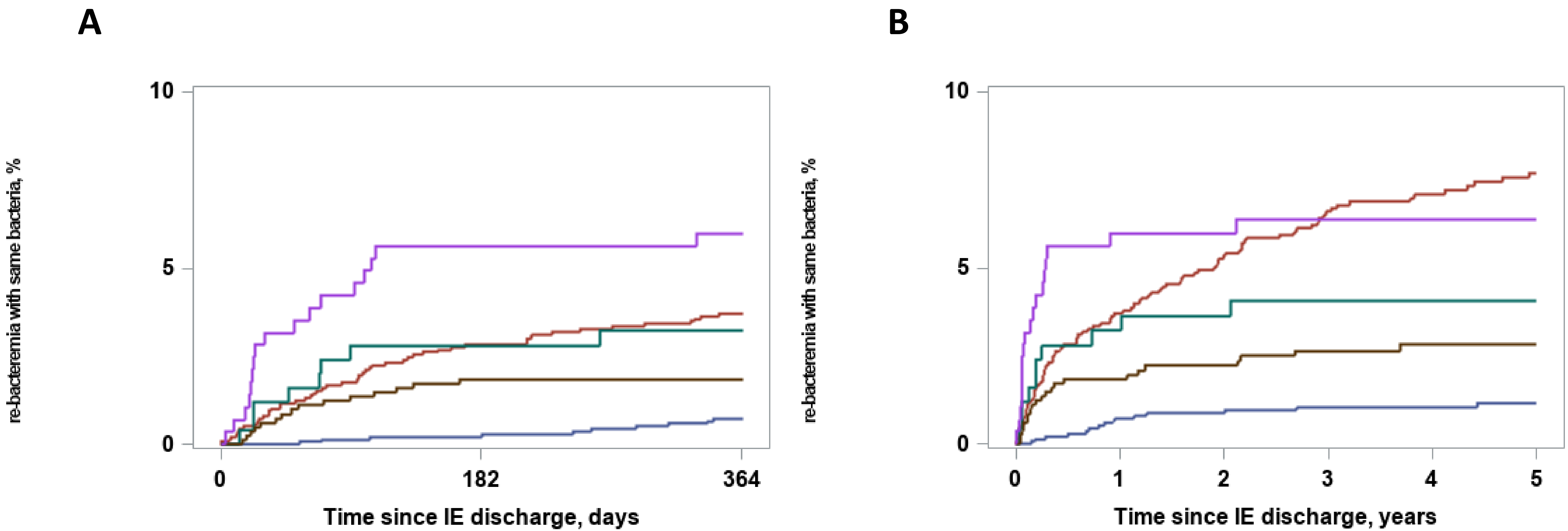

Patients at risk (Panel A)

	0	182	364
Streptococcus	1366	1215	1131
Staphylococcus aureus	1374	1123	1021
Other	249	203	194
Enterococcus	813	646	592
CoNS	284	222	208

Patients at risk (Panel B)

Streptococcus	1366	1215	1131	1023	915	826	731	643	579	500	433
Staphylococcus aureus	1374	1123	1021	891	779	695	602	544	467	409	363
Other	249	203	194	174	159	143	120	105	94	80	74
Enterococcus	813	646	592	517	466	420	370	324	275	224	196
CoNS	284	222	208	191	171	149	133	115	104	88	84

Supplementary Figure 1. The figure shows the cumulative incidence of a recurrent episode of bacteremia (not including IE episodes) with same bacterial species causing the primary episode of IE within 12 months of follow-up, Panel A (left) and a maximum of five years of follow-up, Panel B (right).
