# Supplementary material for: Recurrence of bacteremia and infective endocarditis according to bacterial species of index endocarditis episode
Source: Infection. 2023 Jul 3;51(6):1739–47. doi: 10.1007/s15010-023-02068-x (PMC10665237; doi:10.1007/s15010-023-02068-x)
Supplement: Supplementary file 2 — Supplementary file2 (PDF 133 KB) [file 15010_2023_2068_MOESM2_ESM.pdf]

**Supplementary Figure 2. Cumulative incidence of recurrent bacteremia with the same bacterial species as the primary IE.**

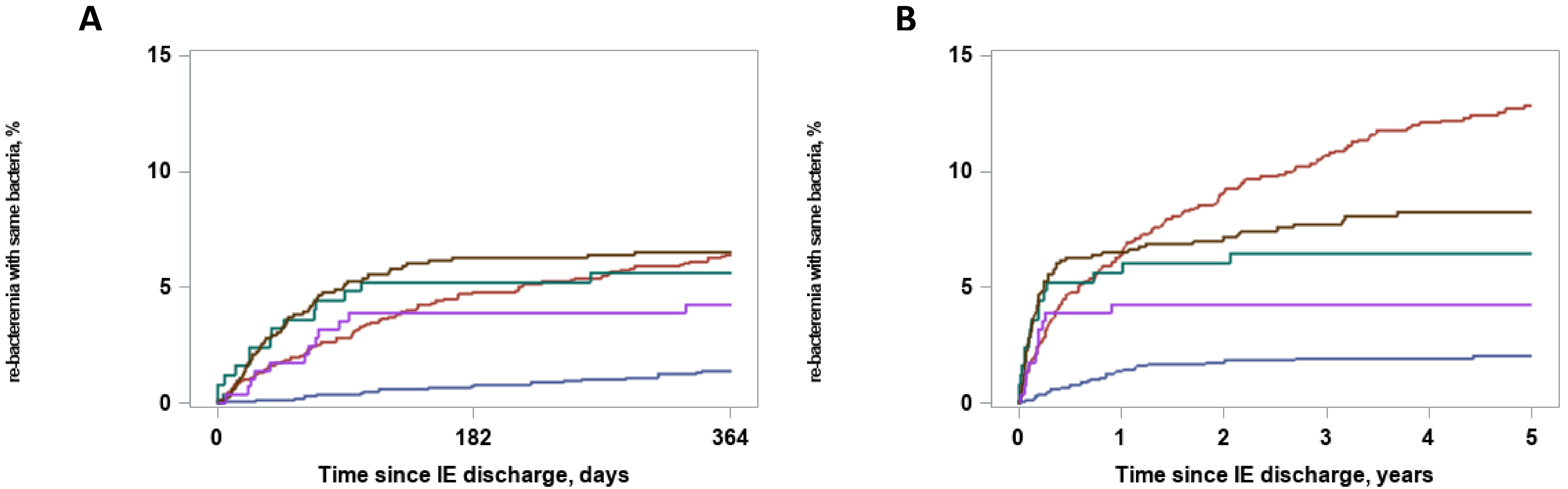


Patients at risk (Panel A)

| | 0 | 182 | 364 |
|---|---|---|---|
| Streptococcus | 1366 | 1225 | 1143 |
| Staphylococcus aureus | 1374 | 1124 | 1022 |
| Other | 249 | 204 | 195 |
| Enterococcus | 813 | 645 | 591 |
| CoNS | 284 | 230 | 216 |

Patients at risk (Panel B)

| | | | | | | | | | | | |
|---|---|---|---|---|---|---|---|---|---|---|---|
| Streptococcus | 1366 | 1225 | 1143 | 1037 | 929 | 839 | 745 | 656 | 591 | 511 | 444 |
| Staphylococcus aureus | 1374 | 1124 | 1022 | 892 | 780 | 696 | 603 | 544 | 467 | 409 | 363 |
| Other | 249 | 204 | 195 | 175 | 161 | 144 | 121 | 106 | 94 | 80 | 74 |
| Enterococcus | 813 | 645 | 591 | 516 | 465 | 419 | 368 | 322 | 273 | 224 | 196 |
| CoNS | 284 | 230 | 216 | 199 | 179 | 158 | 141 | 123 | 112 | 95 | 91 |

Supplementary Figure 2. The figure shows the cumulative incidence of a recurrent episode of bacteremia with same bacterial species causing the primary episode of IE within 12 months of follow-up, Panel A (left) and a maximum of five years of follow-up, Panel B (right). Reccurence of CoNS bacteremia was defined from two positive blood cultures 24 hours apart within 7 days.
