# Supplementary material for: Recurrence of bacteremia and infective endocarditis according to bacterial species of index endocarditis episode
Source: Infection. 2023 Jul 3;51(6):1739–47. doi: 10.1007/s15010-023-02068-x (PMC10665237; doi:10.1007/s15010-023-02068-x)
Supplement: Supplementary file 3 — Supplementary file3 (DOCX 30 KB) [file 15010_2023_2068_MOESM3_ESM.docx]

**Supplementary Tables**

**Recurrence of bacteremia and infective endocarditis according to bacterial pathogen of index endocarditis episode: a nationwide study**

Supplementary Table 1. Codes, p. 2

Supplementary Table 2a. Streptococcus spp. - distribution on species level, p. 3

Supplementary Table 2b. Coagulase negative *staphylococci* - distribution on species level, p. 4

Supplementary Table 2c. Other microbiological etiology - distribution on species level, p. 5

Supplementary Table 3. Microorganisms for recurrent episode of bacteremia and IE, p. 6

| **Supplementary Table 1: Codes** | |
| --- | --- |
| **Category** | **Codes** |
| Study population |  |
| Infective endocarditis | ICD-10: DI33, DI38, DI398; ICD8: 421 (in order to ensure a first-time event) |
|  |  |
| Comorbidity/previous intervention |  |
| Malignancy | ICD10: DC00-DC97, not including C44 (skin cancer) |
| Chronic renal failure | ICD10: DN18-19. |
| Renal dialysis | NOMESCO: BJFD |
| Chronic obstructive lung disease | ICD10: DJ41-44 |
| Heart failure | ICD10: DI42, DI43, DI50, DI110, DI517. |
| Stroke | ICD10: DI61, DI63-64. |
| AMI | ICD10: DI21-22. |
| Prosthetic heart valve | KFKD, KFMD, KFGE, KFJF |
| Atrial flutter/fibrillation | ICD-10: DI48. |
| Alcohol abuse | ICD-10: F10, K70, E52, T51, K860, E244, G312, I426, O354, Z714, Z721, G621, G721, K292, L278A, ICD8: 291, 303, 57109, 57110, 57710. |
| Liver disease | ICD10: B15-19, K70-77, C22, I982, Z944, D684C, Q618A, ICD8: 571-573, 155, 070 |
| PCI | NOMESCO: KFNG00, KFNG02, KFNG05, KFNG10, KFNG12, KFNG96 |
| CABG | NOMESCO: KFNA, KFNB, KFNC, KFNC, KFNE |
| CIED | NOMESCO: BFCA0, BFCB0 |
| Diabetes mellitus | ICD10: E10-14, ATC code: A10 |
|  |  |
| Pharmacotherapy |  |
| Aspirin | ATC code: B01AC06 |
| OAC | ATC code: B01AA03-4, B01AE07, B01AF01, B01AF02 |
| ADPi | ATC code: B01AC04, B01AC22, B01AC24, B01AC25 |
| Statin | ATC code: C10AA |
| Beta blockade | ATC code: C07, C09BX |
| RASi | ATC code: C09 |
| Loop diuretics | ATC code: C03C, C03EB01-2 |
| ICD: international classification of diseases, ATC: Anatomical Therapeutical Classification System, AMI: acute myocardial infarction, PCI: percutaneous coronary intervention, CABG: coronary artery bypass grafting, CIED: cardiac implantable electronic device, OAC: oral anticoagulant therapy, RASi: renin angiotensin system inhibitor, ADPi: adenosine di phosphate inhibitor. | |

| **Supplementary Table 2a. Streptococcus spp. - distribution on species level** | |
| --- | --- |
| *Abiotrophia defectiva* | 22 (1.6) |
| *Gemella haemolysans* | 5 (0.4) |
| *Granulicatella adiacens* | 16 (1.2) |
| *Granulicatella elegans* | 4 (0.3) |
| Hemolytic streptococci group A | 30 (2.2) |
| Hemolytic streptococci group B | 126 (9.2) |
| Hemolytic streptococci group C | 52 (3.8) |
| Hemolytic streptococci group G | 109 (8.0) |
| *Streptococcus anginosus* | 53 (3.9) |
| *Streptococcus bovis* | 99 (7.3) |
| *Streptococcus constellatus* | 4 (0.3) |
| *Streptococcus dysgalactiae* | 26 (1.9) |
| *Streptococcus equinus* | 45 (3.3) |
| *Streptococcus gallolyticus* | 35 (2.6) |
| *Streptococcus gordonii* | 34 (2.5) |
| *Streptococcus infantarius* | 5 (0.4) |
| *Streptococcus intermedius* | 5 (0.4) |
| *Streptococcus milleri* | 9 (0.7) |
| *Streptococcus mitis* | 344 (25.2) |
| *Streptococcus mutans* | 91 (6.7) |
| *Streptococcus oralis* | 31 (2.3) |
| *Streptococcus parasanguinis* | 10 (0.7) |
| *Streptococcus pneumoniae* | 64 (4.7) |
| *Streptococcus salivarius* | 46 (3.4) |
| *Streptococcus sanguinis* | 99 (7.3) |
| Species with less than 3 observations were not reported | |

| **Supplementary Table 2b. Coagulase negative *staphylococci* - distribution on species level** | |
| --- | --- |
| *Staphylococcus capitis* | 18 (6.3) |
| *Staphylococcus epidermidis* | 192 (67.6) |
| *Staphylococcus haemolyticus* | 9 (3.2) |
| *Staphylococcus hominis* | 23 (8.1) |
| *Staphylococcus lugdunensis* | 34 (12.0) |
| *Staphylococcus warneri* | 4 (1.4) |
| Other staphylococci | 4 (1.4) |

| **Supplementary Table 2c. Other microbiological etiology - distribution on species level** | |
| --- | --- |
| *Aerococcus urinae* | 25 (10.0) |
| *Aggregatibacter actinomycetemcomitans* | 16 (6.4) |
| *Aggregatibacter aphrophilus* | 8 (3.2) |
| *Bacteroides fragilis* | 3 (1.2) |
| *Cardiobacterium hominis* | 12 (4.8) |
| *Corynebacterium striatum* | 4 (1.6) |
| *Enterobacter cloacae* | 12 (4.8) |
| *Escherichia coli* | 35 (14.1) |
| *Haemophilus influenzae* | 5 (2.0) |
| *Haemophilus parainfluenzae* | 13 (5.2) |
| *Kingella kingae* | 5 (2.0) |
| *Klebisella oxytoca* | 9 (3.6) |
| *Klebsiella pneumoniae* | 11 (4.4) |
| *Lactobacillus rhamnosus* | 4 (1.6) |
| *Cutibacterium acnes* | 21 (8.4) |
| *Pseudomonas aeruginosa* | 9 (3.6) |
| *Rothia dentocariosa* | 3 (1.2) |
| *Serratia marcescens* | 10 (4.0) |
| Other* | 44 (17.7) |

*was combined as numbers for individual bacteria was less than 3

| **Supplementary Table 3. Microorganisms for recurrent episode of bacteremia and IE** | |
| --- | --- |
| **Recurrent bacteremia with 1 year follow-up** |  |
| *Staphylococcus aureus*, n (%) | 88 (44.4) |
| *Enterococcus faecalis*, n (%) | 44 (22.2) |
| *Staphylococcus epidermidis*, n (%) | 24 (12.1) |
| Other*, n (%) | 10 (5.1) |
| Other *Enterococci spp.**, n (%) | 9 (4.6) |
| *Hemolytic streptococci, Group B*, n (%) | 7 (3.5) |
| *Streptococcus* spp.*, n (%) | 5 (2.5) |
| *Abiotrophia defectiva,* n (%) | 4 (2.0) |
| *Escherichia coli,* n (%) | 4 (2.0) |
| *Hemolytic streptococci, group G,* n (%) | 3 (1.5) |
| Total, N (%) | 198 (100) |
|  |  |
| **Recurrent bacteremia with 5 years follow-up** |  |
| *Staphylococcus aureus*, n (%) | 161 (54.6) |
| *Enterococcus faecalis*, n (%) | 54 (18.3) |
| *CoNS**, n (%) | 26 (8.8) |
| Other *Enterococci spp.**, n (%) | 11 (3.6) |
| Other*, n (%) | 10 (3.4) |
| *Hemolytic streptococci, Group B*, n (%) | 7 (2.4) |
| *Hemolytic streptococci, Group G*, n (%) | 6 (2.0) |
| *Escherichia coli,* n (%) | 6 (2.0) |
| *Streptococcus* spp.*, n (%) | 4 (1.4) |
| *Abiotrophia defectiva,* n (%) | 4 (1.4) |
| *Hemolytic streptococci, Group C*, n (%) | 3 (1.0) |
| *Streptococcus pneumoniae,* n (%) | 3 (1.0) |
| Total, N (%) | 295 (100) |
|  |  |
| **Recurrent IE with 1 year follow-up** |  |
| *Staphylococcus aureus*, n (%) | 42 (39.3) |
| *Enterococcus faecalis*, n (%) | 34 (31.8) |
| *Streptococcus* spp.*, n (%) | 13 (12.1) |
| *Staphylococcus epidermidis*, n (%) | 7 (6.5) |
| Other*, n (%) | 6 (5.6) |
| Other *Enterococci* spp.*, n (%) | 5 (4.7) |
| Total, N (%) | 107 (100) |
|  |  |
| **Recurrent IE with 5 years follow-up** |  |
| *Staphylococcus aureus*, n (%) | 75 (48.4) |
| *Enterococcus faecalis*, n (%) | 40 (25.8) |
| *CoNS**, n (%) | 10 (6.5) |
| *Streptococcus* spp.*, n (%) | 9 (5.8) |
| *Streptococcus, mitis group* | 7 (4.5) |
| Other*, n (%) | 6 (3.9) |
| Other *Enterococci* spp.*, n (%) | 5 (3.2) |
| *Abiotrophia defectiva* | 3 (1.9) |
| Total, N (%) | 155 (100) |
| *Further subclassification was not possible due to rules of anonymization. CoNS: coagulase negative staphylococci | |
